# Supplementary material for: Enhancement of Ambulatory Glucose Profile for Decision Assistance and Treatment Adjustments
Source: Diagnostics (Basel). 2024 Feb 16;14(4):436. doi: 10.3390/diagnostics14040436 (PMC10888350; doi:10.3390/diagnostics14040436)
Supplement: Supplementary file 1 [file diagnostics-14-00436-s001.zip › diagnostics-2803179-supplementary.pdf]

**Supplementary Material 1: Metrics and formulas**

Following are the statistical formulas to evaluate the metrics:

1. Standard deviation rate of change (SDR): The rate of change of the SD is calculated as

$$SDR = \frac{[BG(T_I) - BG(T_{I-1})]}{(T_I - T_{I-1})}$$

where,

$SDR = Standard\ Deviation\ Rate\ of\ Change, BG$= blood glucose levels, $T_I$=Timestamp at $I^{th}$ blood glucose level, $T_{I-1}$ =Timestamp at the ($I$-1)$^{th}$ blood glucose level

2. Interquartile Range (IQR): The Interquartile range was calculated using the following equation:

$$IQR = Third\ quartile(Q3) - first\ quartile(Q1)$$

where,

*Q3 =third quartile, Q1=first quartile*

3. Standard deviation (SD): The SD was calculated as,

$$SD = \sqrt{\frac{\sum_{tn=tn_1}^{tn_N}(BG_{tni} - \overline{BG_{tn}})^2}{N-1}}$$

where,

$SD = Standard\ Deviation, BG_{tn1} =\ individual\ BG\ observation,$

$\overline{BG_{tn}}\ = Mean\ of\ BG\ observations$

4. Mean of daily differences (MODD): A higher MODD indicated an irregular lifestyle. MODD is calculated as,

$$MODD = \frac{\sum_{tn-tn_1}^{tn_P}|BG_{tn} - BG_{tn-1440}|}{P}$$

where,

$MODD = Mean\ of\ daily\ differences,$ *tn=timestamp,* $tn_1, tn_P$ *=timestamp at 1st cgm reading and last reading,* $BG_{tn}$ *=individual cgm readings,* $BG_{1440}$*= mean of cgm readings for 24 hours, N=number of cgm readings*

5. Continuous Overall Net Glycemic Action (CONGA): CONGA was calculated as,

$$CONGA = \sqrt{\frac{\sum_{tn=tn_1}^{tn_P}(D_{tn} - \overline{D})^2}{P-1}}$$

where,

CONGA = $Continuous\ Overall\ Net\ Glycemic\ Action, D_{tn} = BG_{tn} - BG_{tn-M}$ , $\overline{D} = \frac{\sum_{tn-tn_1}^{tnP} SD_{tn}}{P}$ , $D_{tn}$ =difference between BG taken at time t and BG taken n h earlier-, $\overline{D}$ =average of these ($D_{tn}$ )differences, $BG_{tn} - BG_{tn-M}$ , P= number of available BG observations in 24 h (1440min) apart, $M$=n×60

6. Mean amplitude of glycemic excursions (MAGE): MAGE is calculated as,

$$MAGE = \frac{\sum \alpha}{Y} \text{ if } \alpha > SD$$

where,

MAGE = Mean Amplitude of Glycemic Excursions ,$\alpha$=absolute difference between sequential glucose peaks and nadirs, $Y$ =no. of valid observations,$\beta$ =1 SD of the mean glucose level.

**Web Interface**

1(A)

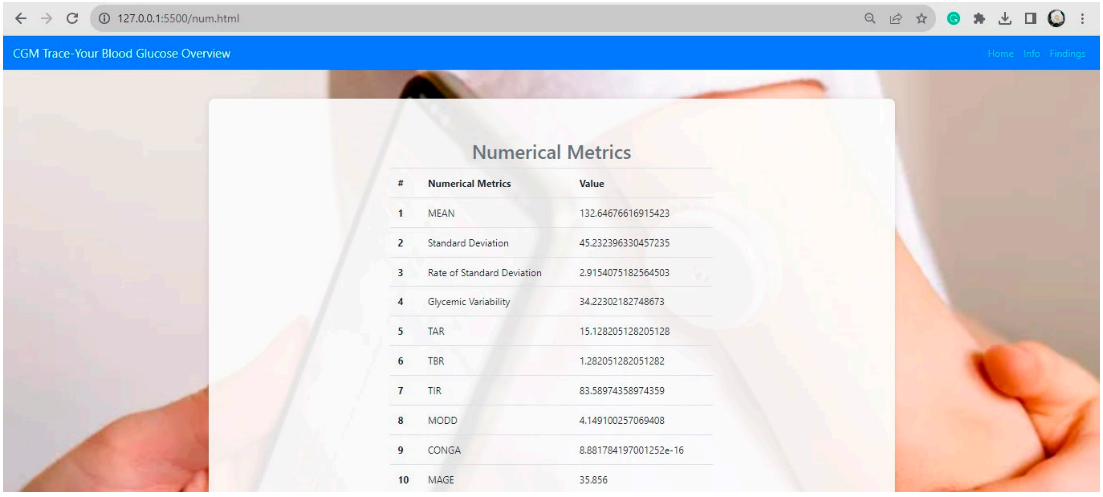


1(B)

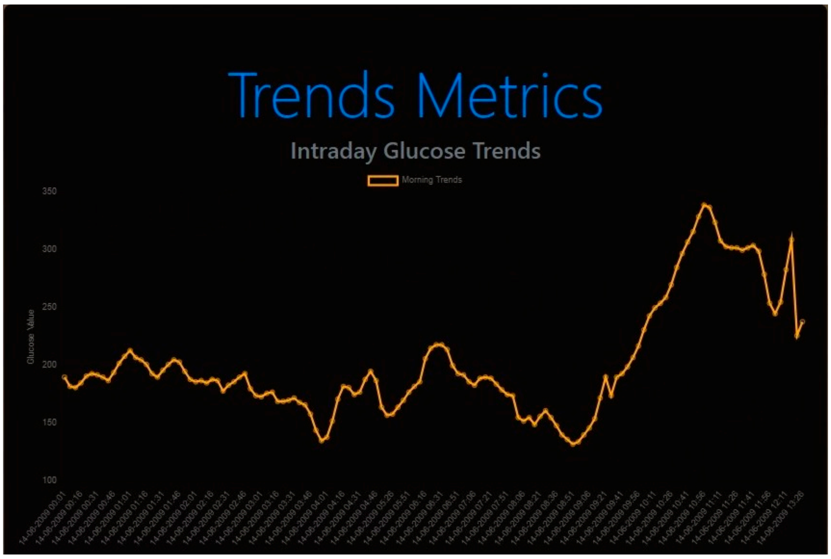

1(C)

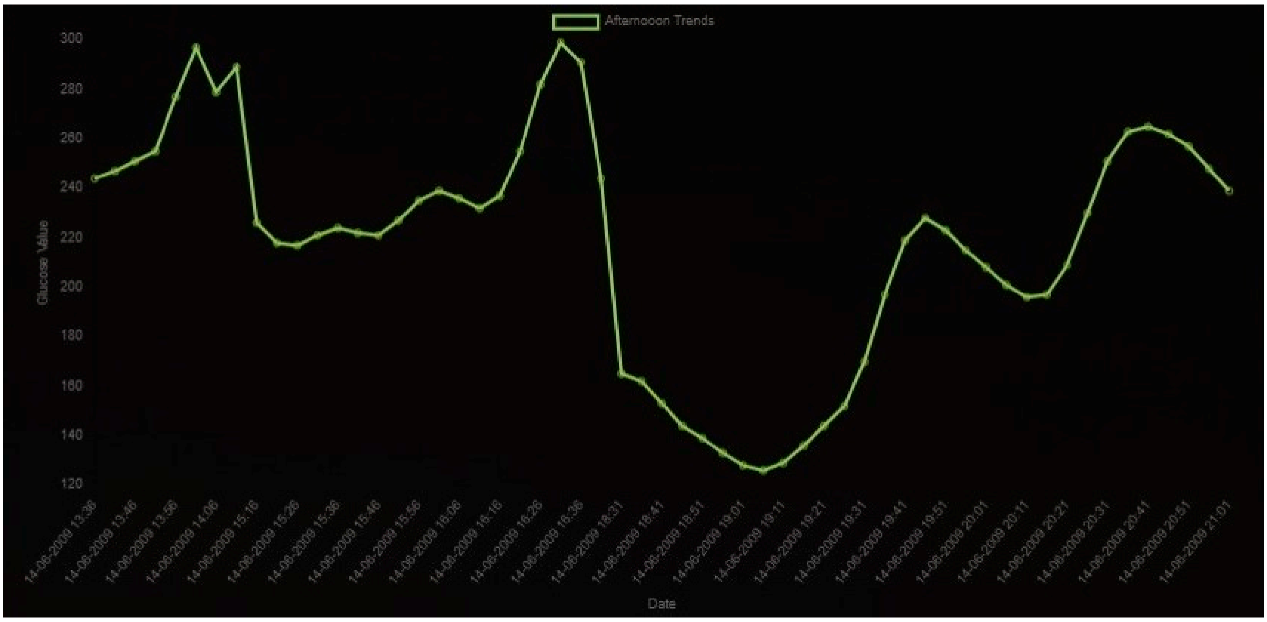
Afternooon Trends
Glucose Value
300
280
260
240
220
200
180
160
140
120
14-06-2009 13:36
14-06-2009 13:46
14-06-2009 13:56
14-06-2009 14:06
14-06-2009 15:16
14-06-2009 15:26
14-06-2009 15:36
14-06-2009 15:46
14-06-2009 15:56
14-06-2009 16:06
14-06-2009 16:18
14-06-2009 16:26
14-06-2009 16:36
14-06-2009 18:31
14-06-2009 18:41
14-06-2009 18:51
14-06-2009 19:01
14-06-2009 19:11
14-06-2009 19:21
14-06-2009 19:31
14-06-2009 19:41
14-06-2009 19:51
14-06-2009 20:01
14-06-2009 20:11
14-06-2009 20:21
14-06-2009 20:31
14-06-2009 20:41
14-06-2009 20:51
14-06-2009 21:01
Date

1(D)

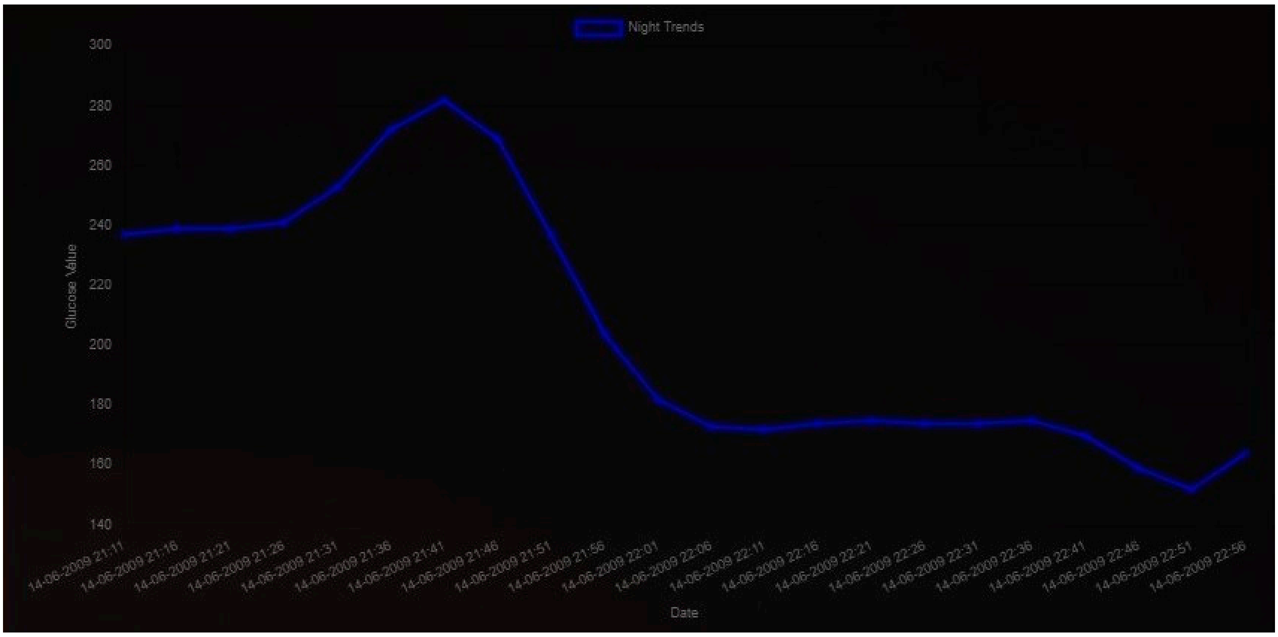
Night Trends
Glucose Value
300
280
260
240
220
200
180
160
140
14-06-2009 21:11
14-06-2009 21:16
14-06-2009 21:21
14-06-2009 21:26
14-06-2009 21:31
14-06-2009 21:36
14-06-2009 21:41
14-06-2009 21:46
14-06-2009 21:51
14-06-2009 21:56
14-06-2009 22:01
14-06-2009 22:06
14-06-2009 22:11
14-06-2009 22:16
14-06-2009 22:21
14-06-2009 22:26
14-06-2009 22:31
14-06-2009 22:36
14-06-2009 22:41
14-06-2009 22:46
14-06-2009 22:51
14-06-2009 22:56
Date

1(E)

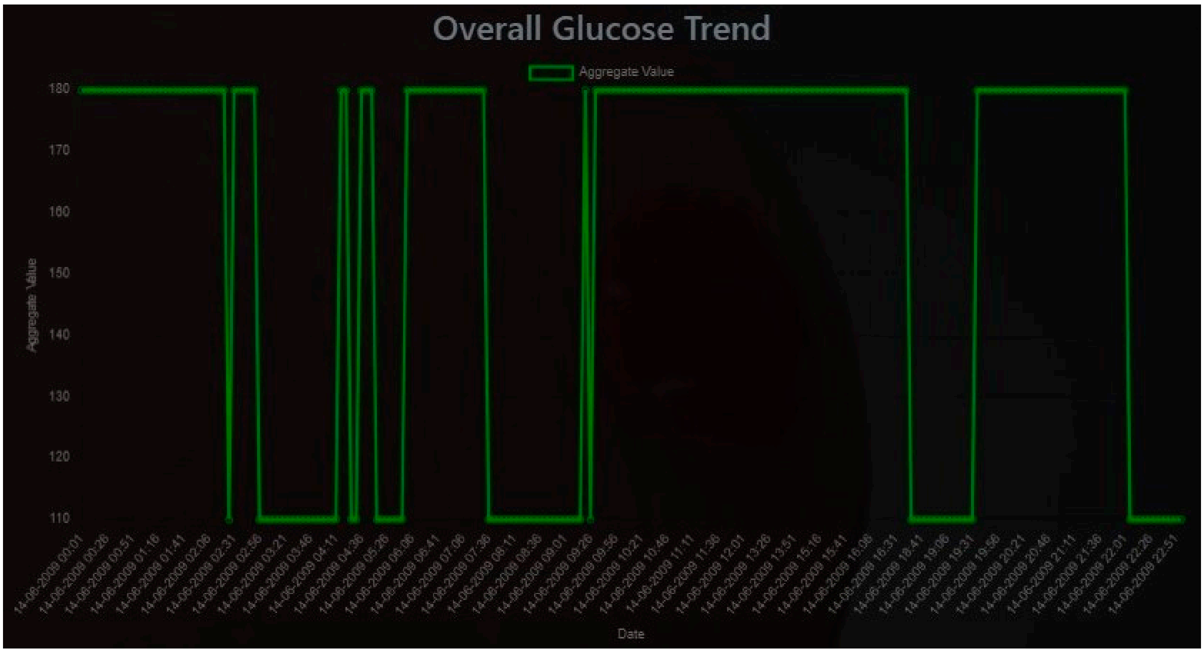
Overall Glucose Trend
Aggregate Value
Aggregate Value
Date

1(F)

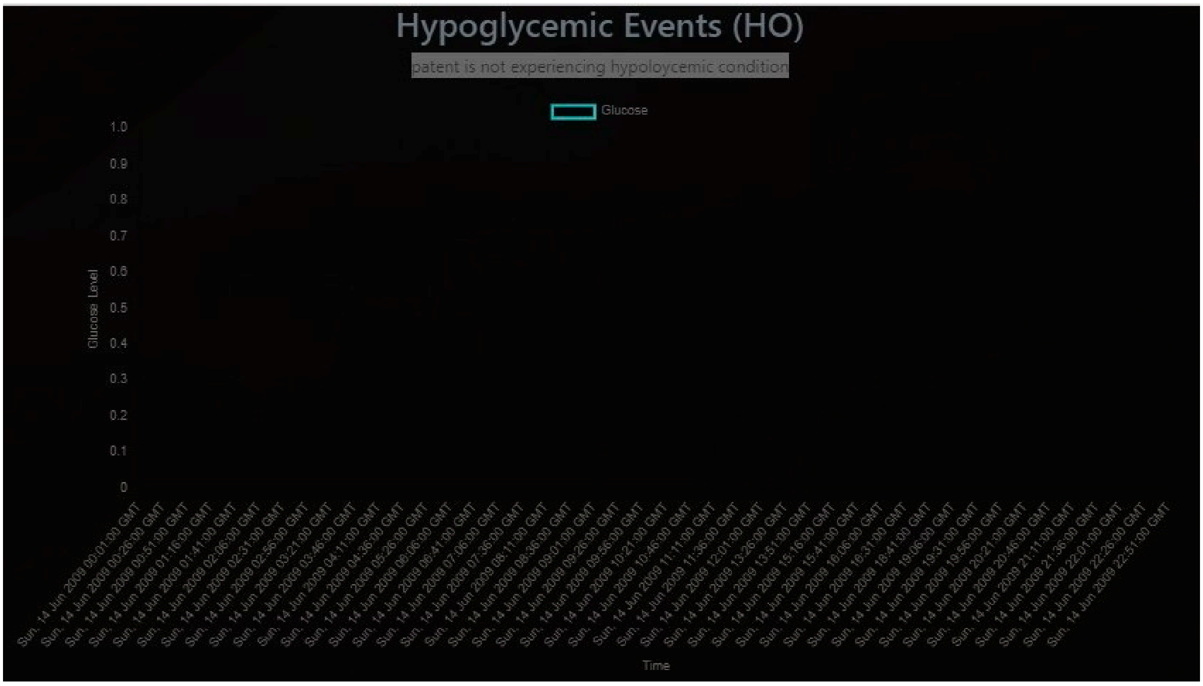
Hypoglycemic Events (HO)
patent is not experiencing hypoloycemic condition
Glucose
Glucose Level
Time

1(G)

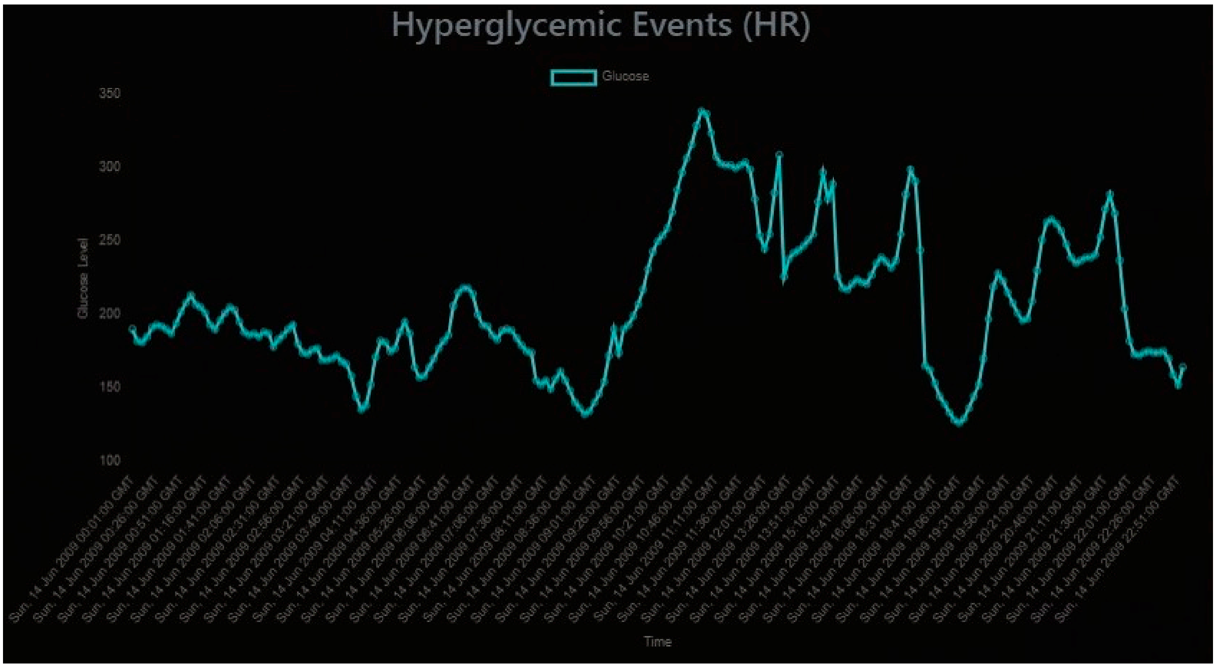


1(H)

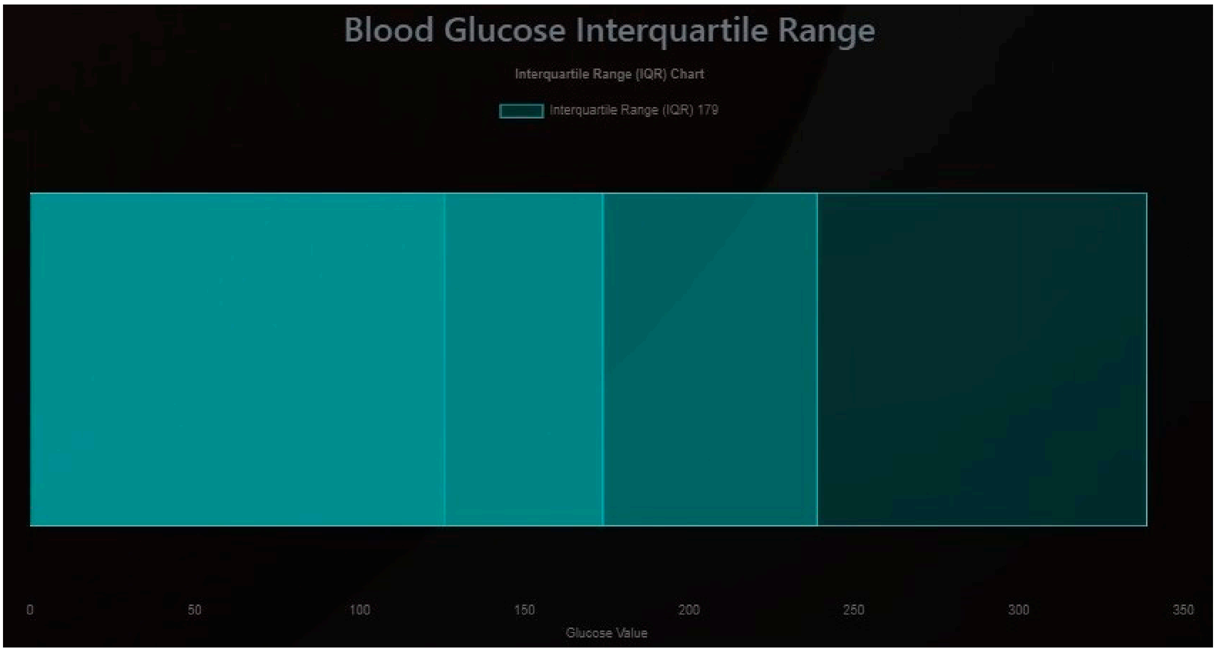


Supplementary Figure S1. Display of clinical metrics and respective graphs. (a) Display page of numerical metrics (b) Intraday Glucose Trends-morning (c) Intraday Glucose Trends- afternoon (d) Intraday Glucose Trends-midnight (e)Overall glucose trends (f) Hypoglycaemic trends (g)Hyperglycaemic trends (h) IQR graph

**Table S1** CGM data analysis from 'CGM Trace'- Public dataset from JCHR-JAEB

| P | Metrics | | | | | | | | | | | |
|---|---|---|---|---|---|---|---|---|---|---|---|---|
| | **SDR** | **MG** | **SD** | **GV (%)** | **TIR (%)** | **TAR-I (%)** | **TAR-II (%)** | **TBR-I (%)** | **TBR-II (%)** | **MODD** | **CONGA** | **MAGE** |
| 1 | 5.4 | 195 | 66.3 | 42.7 | 62.3 | 30.5 | 5.6 | 0 | 2.0 | 5.6 | $4.8e^{-16}$ | 52.2 |
| 2 | 5.3 | 183 | 118 | 64.7 | 52.6 | 12.7 | 26.0 | 5.2 | 2.3 | 5.0 | 0 | 76.4 |
| 3 | 5.1 | 184.6 | 71.5 | 38.7 | 45.0 | 32.8 | 19.7 | 19 | 0 | 6.8 | $8.8e^{-16}$ | 46.1 |
| 4 | 4.0 | 127.7 | 37.9 | 29.7 | 90.4 | 7.7 | 0 | 1.4 | 0.7 | 5.8 | $8.4e^{-16}$ | 21.6 |
| 5 | 3.2 | 168.9 | 66.8 | 39.4 | 70.7 | 20.0 | 8.3 | 0.7 | 0 | 7.4 | $8.2e^{-16}$ | 49.3 |
| 6 | 3.3 | 142.8 | 54.6 | 38.2 | 68.2 | 25.0 | 4.7 | 3.1 | 0 | 4.78 | 0 | 20.8 |
| 7 | 3.2 | 147.7 | 66.9 | 45.2 | 22.2 | 71.2 | 0 | 6.5 | 0 | 3.7 | $7.9e^{-16}$ | 36.8 |
| 8 | 4.0 | 130.8 | 46 | 35.1 | 80.2 | 10.0 | 3.9 | 3.6 | 2.6 | 5.6 | $7.8e^{-16}$ | 25 |
| 9 | 5.3 | 135.8 | 53.8 | 39.6 | 70.0 | 19.5 | 1.9 | 5.3 | 2.6 | 7.9 | 0 | 31.5 |
| 10 | 3.7 | 157 | 46.5 | 29.5 | 78.1 | 16.5 | 4.5 | 0 | 0 | 3.75 | 0 | 33.7 |
| 11 | 4.0 | 157.8 | 47.2 | 29.9 | 62.7 | 31.1 | 7.0 | 0 | 0 | 4.5 | $7.8e^{-16}$ | 44.9 |
| 12 | 6.2 | 128.7 | 39.5 | 29.5 | 89.8 | 5.7 | 0 | 4.5 | 1.4 | 5.4 | $9.8e^{-16}$ | 34 |
| 13 | 3.1 | 116.4 | 38.6 | 33.1 | 92.7 | 4.5 | 2.1 | 0.4 | 0 | 4.5 | $3.8e^{-16}$ | 27.2 |
| 14 | 2.7 | 120.4 | 49.4 | 40.9 | 77.8 | 7.3 | 2.9 | 4.4 | 7.9 | 3.6 | 0 | 24.8 |
| 15 | 2.5 | 170 | 70.6 | 31.5 | 39.4 | 62.2 | 17.5 | 20.1 | 0 | 3.1 | 0 | 93.1 |
| 16 | 5.4 | 121.9 | 43.7 | 35.8 | 68.4 | 18.5 | 0.4 | 10.7 | 1.6 | 3.4 | 0 | 30 |
| 17 | 5.5 | 176.2 | 50 | 28.3 | 58.6 | 33.5 | 7.1 | 1.2 | 0 | 6.3 | 0 | 26.6 |
| 18 | 4.4 | 129.8 | 44 | 38.8 | 80.8 | 13.0 | 0 | 5.1 | 1.4 | 6.3 | $1.7e^{-16}$ | 13.6 |
| 19 | 3.6 | 147.1 | 53.9 | 36.6 | 77.8 | 12.3 | 9.4 | 0.9 | 0 | 5.9 | $5.8e^{-16}$ | 29.8 |
| 20 | 7 | 187.1 | 66.3 | 36.6 | 52.7 | 31.7 | 16.9 | 0 | 0 | 5.1 | $4.8e^{-16}$ | 29.8 |
| 21 | 2.7 | 158.4 | 39.9 | 25.1 | 74.7 | 25.0 | 0 | 0 | 0 | 5.1 | $7.8e^{-16}$ | 37.54 |
| 22 | 3.3 | 194.7 | 65.9 | 38.8 | 63.7 | 43.7 | 20.3 | 1.7 | 0.7 | 4.4 | $6.5e^{-16}$ | 48.6 |
| 23 | 7.3 | 163.6 | 74.8 | 45.6 | 55.4 | 24.7 | 12.0 | 5.6 | 1.7 | 8.5 | 0 | 52.4 |
| 24 | 3.1 | 203.9 | 50.7 | 24.7 | 35.9 | 53.9 | 26.1 | 0 | 2 | 3.9 | 0 | 41.7 |
| 25 | 3.7 | 227.5 | 86.1 | 34.8 | 30.1 | 25.4 | 42.6 | 1.3 | 1.1 | 5.5 | $7.9e^{-16}$ | 44.8 |
| 26 | 2.5 | 155.2 | 45.3 | 29.1 | 33.7 | 50.5 | 10.9 | 3.8 | 0.2 | 5.8 | $8.8e^{-16}$ | 51.3 |
| 27 | 2.5 | 210 | 51.8 | 24.6 | 27 | 51.2 | 20.8 | 0 | 0 | 3.2 | 0 | 60.7 |
| 28 | 5.9 | 176.2 | 48.4 | 27.4 | 54.6 | 35.4 | 8.8 | 0.9 | 0 | 7.6 | 0 | 11.8 |
| 29 | 6.2 | 151.6 | 65.3 | 33 | 49.3 | 26.0 | 24.3 | 0.2 | 0 | 4.1 | $8.6e^{-16}$ | 41.2 |
| 30 | 3.1 | 230.8 | 43.9 | 19.0 | 17.6 | 44.9 | 36.8 | 0 | 0 | 7.6 | $9.8e^{-16}$ | 54 |
| 31 | 6.3 | 167.2 | 50.0 | 29.8 | 66.1 | 25.8 | 8.0 | 0 | 0 | 8.5 | $7.7e^{-16}$ | 171.4 |
| 32 | 2.9 | 135 | 45.8 | 33.9 | 66.1 | 24 | 2.2 | 5.9 | 1.8 | 3.5 | $6.6e^{-16}$ | 40.22 |
| 33 | 1.9 | 159.3 | 42.9 | 26.9 | 78.1 | 15.9 | 5.8 | 0 | 0 | 2.9 | $4.4e^{-16}$ | 64 |
| 34 | 2.3 | 110.9 | 42.9 | 36 | 81.0 | 7.4 | 1.2 | 10 | 0.76 | 3.31 | $9.7e^{-16}$ | 33.9 |
| 35 | 5.4 | 138.9 | 56.2 | 30.4 | 76.1 | 12.6 | 4.7 | 6.4 | 0.9 | 5.2 | $6.5e^{-16}$ | 23 |
| 36 | 3 | 133.8 | 44.6 | 33.2 | 84.0 | 11.7 | 2.1 | 1.4 | 0 | 4.4 | 0 | 36 |
| 37 | 4.02 | 189.3 | 59.1 | 37.2 | 51.1 | 30.3 | 18.8 | 0 | 0 | 2.7 | $8.2e^{-16}$ | 34.6 |

**Abbreviations**: M,metrics; P,patient; SDR, standard deviation rate of change; SD, Standard deviation; MG, mean glucose; GV, glucose variability; TIR, time in range; TAR, time above range; TBR, time below range; MODD, mean of daily differences; CONGA, continuous overall net glycemic action; MAGE, mean amplitude of glycemic excursions
